# Underwater target laser polarization suppression scattering detection technology and verification

**DOI:** 10.1371/journal.pone.0305929

**Published:** 2024-06-25

**Authors:** Qiang Fu, Chao Dong, Kaikai Wang, Qingyi He, Xiansong Gu, Jianhua Liu, Yong Zhu, Jin Duan

**Affiliations:** 1 College of Opto-Electronic Engineering, Changchun University of Science and Technology, Changchun, China; 2 National and Local Joint Engineering Research Center of Space Optoelectronics Technology, Changchun University of Science and Technology, Changchun, China; 3 Science and Technology Branch, Changchun University of Science and Technology, Changchun, China; 4 College of Computer Science, Changchun University of Science and Technology, Changchun, China; 5 College of Telecommunications, Changchun University of Science and Technology, Changchun, China; Whale Wave Technology Inc, CHINA

## Abstract

The underwater laser polarization detection technology integrates the polarization characteristics of light into the detection and identification of underwater targets. Addressing the challenge of poor accuracy in identifying targets in strong underwater scattering environments, this article proposes an overall scheme for a laser polarization underwater detection device that suppresses scatter using polarized pulse signals. By overcoming key technological barriers in the design of polarization-preserving optical detection systems and utilizing the method of differential amplitude to measure polarization, a laser polarization underwater detection device was developed and underwater polarization detection experiments were conducted, achieving precise detection of underwater targets. The results indicate that the underwater detection device we designed has a root mean square error of less than 5.7% to detect the polarization of the target, demonstrating the accuracy and precision of the underwater detection device.

## Introduction

The ocean is an essential component of the ecosystem on which humans rely for survival. The vast marine areas contain abundant marine dynamics, marine life, seabed minerals, oceanic chemistry, and other resources. The development and protection of these resources has garnered widespread attention from nations and people around the world. In early laser detection systems, the identification of objects relied primarily on the intensity difference between the target and the background. However, in underwater detection, the complex underwater environment, with its high concentration of trace elements, microorganisms, and impurities, leads to stronger scattering and attenuation compared to the transmission of laser beams in the atmosphere. This results in minimal differences in light intensity between the target and the background. Moreover, as the depth increases, energy loss becomes more significant, and the detectable light intensity becomes weaker. Therefore, reliance solely on intensity for identification has certain technical limitations. With the advancement of scientific research, the polarization properties of light have been discovered. Laser polarization detection, a new laser detection technique, has been proposed, serving as a powerful auxiliary means for underwater target detection and offering vast application opportunities.

Researchers have made significant contributions to the detection of underwater polarization imaging detection [1–5]. In recent years, rapid advancements in laser technology have drawn global attention to underwater laser target detection, with many countries conducting extensive research and experiments in this area. Research on laser underwater target detection technology in foreign countries started early and has matured. In the late 1960s, Hickman and Hogg from Syracuse University in the United States developed the first laser seawater detection system, using it to verify the feasibility of laser depth measurement and laying the initial theoretical and experimental foundations for underwater laser detection technology [6]. In 2012, Canada’s Optech Corporation successfully developed the CZM airborne laser depth measurement system, a new generation of deep water and coastal terrain detection systems characterized by high spatial resolution, large optical aperture, and adaptability to poor water quality [7]. In 2021, Li et al. [8] developed a modulated subnanosecond laser radar for underwater target detection, capable of emitting stable and powerful modulated signals, greatly extending the range of underwater detection. The research trend of underwater target laser polarization detection technology is constantly developing and evolving. The research directions of this technology mainly include the following aspects:1)Improving resolution and sensitivity: Researchers are committed to developing new lasers and detectors to enhance the resolution and sensitivity of underwater target laser polarization detection systems, enabling them to detect and identify underwater targets more accurately. In 2021, Shi et al. [9] developed a new type of fiber Bragg grating hydrophone which can detect low frequency signals. The hydrogel and fiber grating are combined in structure, and a combined packaging structure is designed, which can effectively improve the low frequency sensitivity of the hydrophone. But their detection distance is only 4m, in this article we not only improved the detection sensitivity but also increased the detection distance to 3-10m. 2) Multi mode integration: Integrating laser polarization detection technology with other sensor technologies (such as sonar, radar, etc.) to achieve multi-mode underwater target detection and recognition, and improve the all-weather performance of the system. 2020 M Darwiesh et al. [10] proposed a LiDAR model for underwater target detection, which enables users to identify and locate underwater targets with measurable light reflectivity. But they only conducted modeling and simulation analysis without combining experimental verification of the feasibility of the method. 3) Deep learning and artificial intelligence: Utilizing deep learning and artificial intelligence technologies to process and analyze underwater target laser polarization detection data, achieving automatic target detection, recognition, and tracking, improving the intelligence level and real-time performance of the system. The deep learning network model proposed by Wang et al. [11] in 2024 has made significant improvements in object recognition, with an average accuracy (mAP) of over 60% in object detection capability. However, they did not consider issues such as high underwater noise and slow response to laser transmission losses. Therefore, this article proposes to design a signal conditioning circuit that can suppress noise and respond quickly to meet the requirements of subsequent systems. Building on these achievements, this study incorporates the polarization characteristics of light into the detection and identification of underwater targets, conducts research on underwater target polarization imaging technology, proposes an overall scheme for a laser polarization underwater detection device that suppresses water scattering with polarized pulse signals, develops a laser polarization underwater detection device combined with key optical detection system design technologies, and conducts underwater polarization detection experiments, demonstrating that the designed underwater detection device can achieve precise underwater target detection.

Laser underwater detection [12] technology has emerged in recent years as a new remote sensing technology for oceans and rivers. It is one of the research hotspots in the field of underwater target detection technology in China, mainly applied to marine mapping, underwater engineering monitoring, river channel monitoring and early warning, underwater geological disaster early warning, and underwater target detection. Considering the detection of oceanic domains, the development of marine resources, and various other perspectives, strengthening research on laser underwater target detection technology has strategic and significant economic value for national development. Therefore, the development and application of a laser polarization underwater detection system is of pivotal significance for the development and safety of the ocean and rivers. The development and application of this technology are expected to evolve into a novel high-resolution, high-sensitivity, high-efficiency, low-cost underwater target detection and identification technology, providing a new mapping method for global marine mapping, underwater engineering monitoring, river channel monitoring, and more.

## Principle of laser polarization for underwater target detection

### Principle of underwater laser target detection

Laser detection technology is an active detection technique [13], essentially a type of laser radar (Lidar—Light Detection and Ranging). It is a technology that is used to detect, locate, and identify targets submerged in water using a laser for search and tracking. The laser, as the light source of the detection system, possesses advantages that are difficult to compare with other detection systems. Compared to passive detection systems, this type of detection technology has characteristics such as flexible detection, rapid response, easier target identification, good concealment, and high detection accuracy.

Laser detection can be divided into two categories according to the detection mode: optical heterodyne detection (also known as coherent detection) and direct detection (also known as energy detection) [14]. Optical heterodyne detection requires high-performance measurement of two signals simultaneously and the use of two optical paths, which requires more complex instruments and calibration steps. Therefore, we adopt the direct detection method for laser detection.

**Fig 1:**
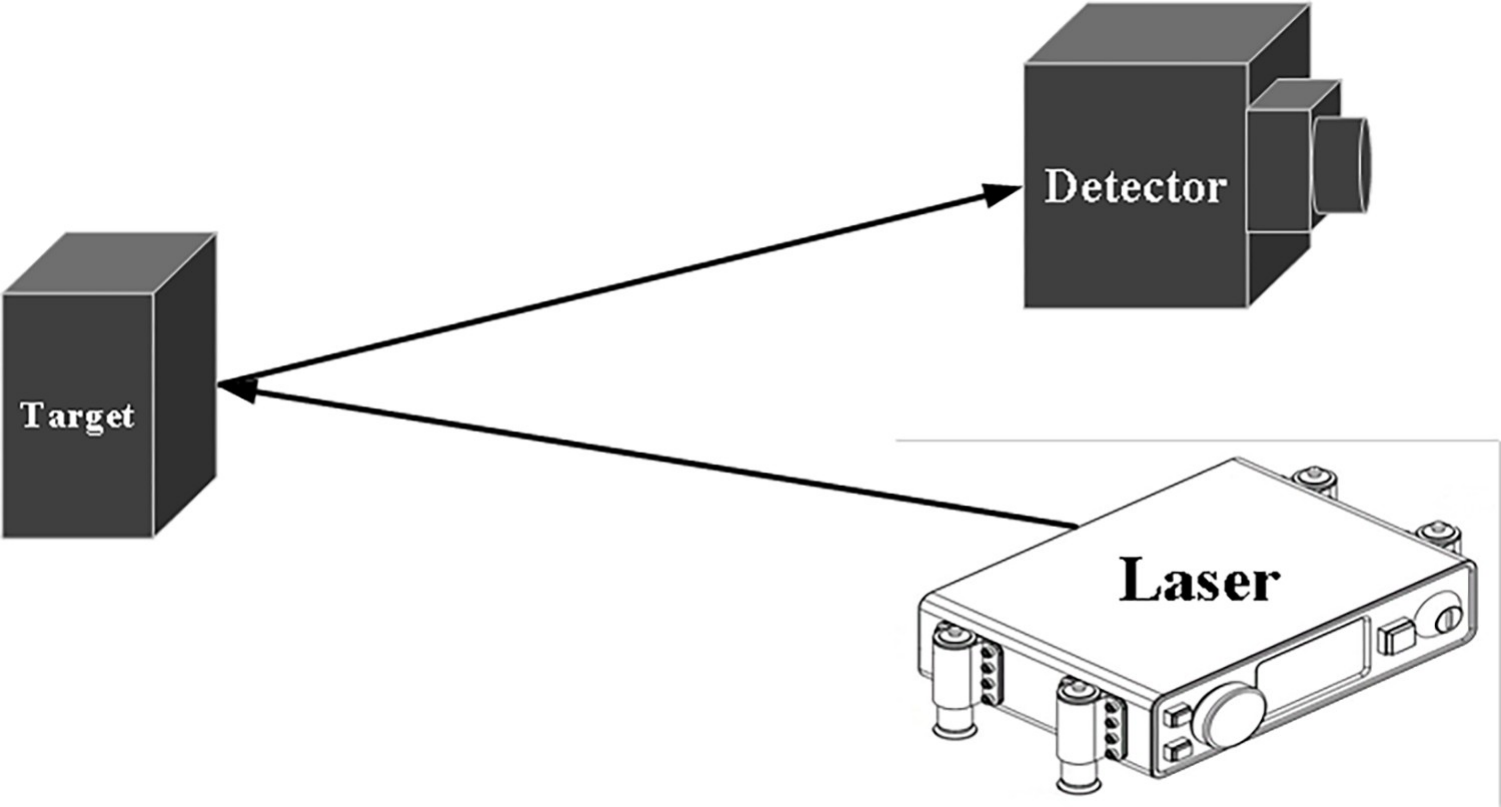
Schematic diagram of the direct detection principle.

The basic principle of direct detection is to utilize photodetector components to respond directly to the changes in the intensity of the laser echo signal, converting the echo signal into a corresponding current or voltage change signal [15]. As shown in Fig 1, the direct detection principle involves the laser signal emitted by the laser reaching the photosensitive surface of the photodetector after reflecting off the target, with the photodetector directly converting the light intensity signal into a corresponding current or voltage signal.

Let the scalar of the signal light field be as follows:

$$E_{s}=A_{s}\cos(\omega_{s}t-\phi_{s})$$ (1)

Where, *A*_*s*_ is the complex amplitude of the signal light, *ω* is the angular frequency of the signal light, and *ϕ* is the initial phase of the signal light. Then the average power of the light on the photosensitive surface of the photodetector is the following.


$$P=\bar{E_{s}^{2}(t)}=A_{s}^{2}\bar{\cos^{2}(\omega_{s}t+\phi_{s})}$$ (2)

Therefore, the photocurrent output photocurrent is:

$$I_{P}=\beta \cdot P=\beta \cdot A_{s}^{2}\bar{\cos^{2}(\omega_{s}t+\phi_{s})}$$ (3)

*P* is the current conversion coefficient of the photodetector. According to Eq (3), the photocurrent of the photodetector is directly proportional to the amplitude of the signal light, which is proportional to the intensity of the signal light. It only contains information about the intensity of the signal light.

Although the optical heterodyne detection mode provides rich information and has a higher signal-to-noise ratio under high-noise background conditions, it requires the use of a single-frequency, high-stability local oscillator laser. This is relatively easier to achieve with gas lasers but more challenging for solid-state lasers. Furthermore, optical heterodyne detection requires a very strict spatio-temporal relationship between the received signal light and the local oscillator light, which limits the reception field and greatly reduces the received optical power.

In comparison, direct detection is a practical, simple, cost-effective, and reliable detection method widely used in underwater laser target detection.

### Principle of polarization target detection

Polarized target detection technology is a relatively new detection technique that utilizes polarization to obtain target polarization signature information, allowing for target detection and identification. Compared to traditional detection methods, polarized detection significantly increases the amount of information about the characteristics of the target, thus improving the accuracy of target detection. Currently, polarized detection has wide-ranging applications in target detection, camouflage recognition, atmospheric detection, remote detection, and other fields [16, 17].

Polarization is an intrinsic property of light, representing the spatial distribution of the electric vector vibration of light waves relative to the direction of light propagation. The loss of symmetry in the spatial distribution of the electric-vector vibration is known as polarization. Through continuous research on polarization phenomena, polarization has gradually been recognized as an important source of information for analyzing the properties of targets.

The British physicist Stokes developed the Stokes vector method while studying polarized light, which utilizes four parameters to describe all states of polarized light. This allows the polarization state and intensity of light waves to be represented by the Stokes vector [18]. The definition of the Stokes vector (*I*,*Q*,*U*,*V*)^*T*^ is shown in Eq (4):

$$S=[\begin{array}{c} I\\ Q\\ U\\ V \end{array}]=[\begin{array}{c} S_{0}\\ S_{1}\\ S_{2}\\ S_{3} \end{array}]=[\begin{array}{c} I(0^{{}^{\circ}})+I(90^{{}^{\circ}})\\ I(0^{{}^{\circ}})-I(90^{{}^{\circ}})\\ I(45^{{}^{\circ}})-I(135^{{}^{\circ}})\\ I_{r}-I_{l} \end{array}]$$ (4)

The intensity, polarization state, and phase difference of polarized light can all be represented using the Stokes vector. The four components of the Stokes *I*,*Q*,*U*,*V* vector can represent fully polarized light, partially polarized light, and unpolarized light. In Eq (4), the total intensity is represented by *I*, the difference in the intensity components between the linear polarization is represented by *Q*, the difference in intensity components between 45° and 135° linear polarization is represented by *U*, and the difference in intensity components between the circular polarization left and right is represented by *V*. Here, $I(0^{{}^{\circ}})、I(45^{{}^{\circ}})、I(90^{{}^{\circ}})、I(135^{{}^{\circ}})$ represents the intensity value of linear polarized light at 0°, 45°, 90° and 135°. *I*_*r*_ represents the intensity value of the right-handed circular polarized light, and *I*_*l*_ represents the intensity value of the left-handed circular polarized light.

Using the four Stokes parameters of Eq (4), one can obtain polarization information. By processing and calculating the four parameters *I*,*Q*,*U*,*V* numerical values can be derived for degree of Linear polarization (DOLP), Degree of Polarization (DOP), and Angle of Polarization (AOP) can be derived [19]. These values provide insights into the characteristics of polarized light, making it more convenient to study the transmission properties of underwater polarized information. Once the Stokes vector is obtained, Eqs (5), (6), and (7) can be used to calculate the numerical values for DOLP, DOP, and AOP.


$$DOLP=\frac{\sqrt{Q^{2}+U^{2}}}{I}$$ (5)

$$DOP=\frac{\sqrt{Q^{2}+U^{2}+V^{2}}}{I}$$ (6)

$$AOP=\frac{1}{2}Arc\tan(\frac{U}{Q})$$ (7)

Due to the minimal and difficult-to-measure circular polarization component (*V*) of targets in the natural world, the magnitude of the circular polarization component can be considered negligible compared to measurement equipment errors. It is common practice to set *V = 0*. Therefore, to fully represent the polarization state of a beam of light, only the three Stokes vector parameters, *I*, *Q*, and *V*, are needed.


$$DOP=\frac{\sqrt{Q^{2}+U^{2}}}{I}=\frac{\sqrt{{\left(I_{0^{{}^{\circ}}}-I_{90^{{}^{\circ}}}\right)}^{2}+{\left(I_{45^{{}^{\circ}}}-I_{135^{{}^{\circ}}}\right)}^{2}}}{I_{0^{{}^{\circ}}}+I_{90^{{}^{\circ}}}}$$ (8)

**Fig 2:**
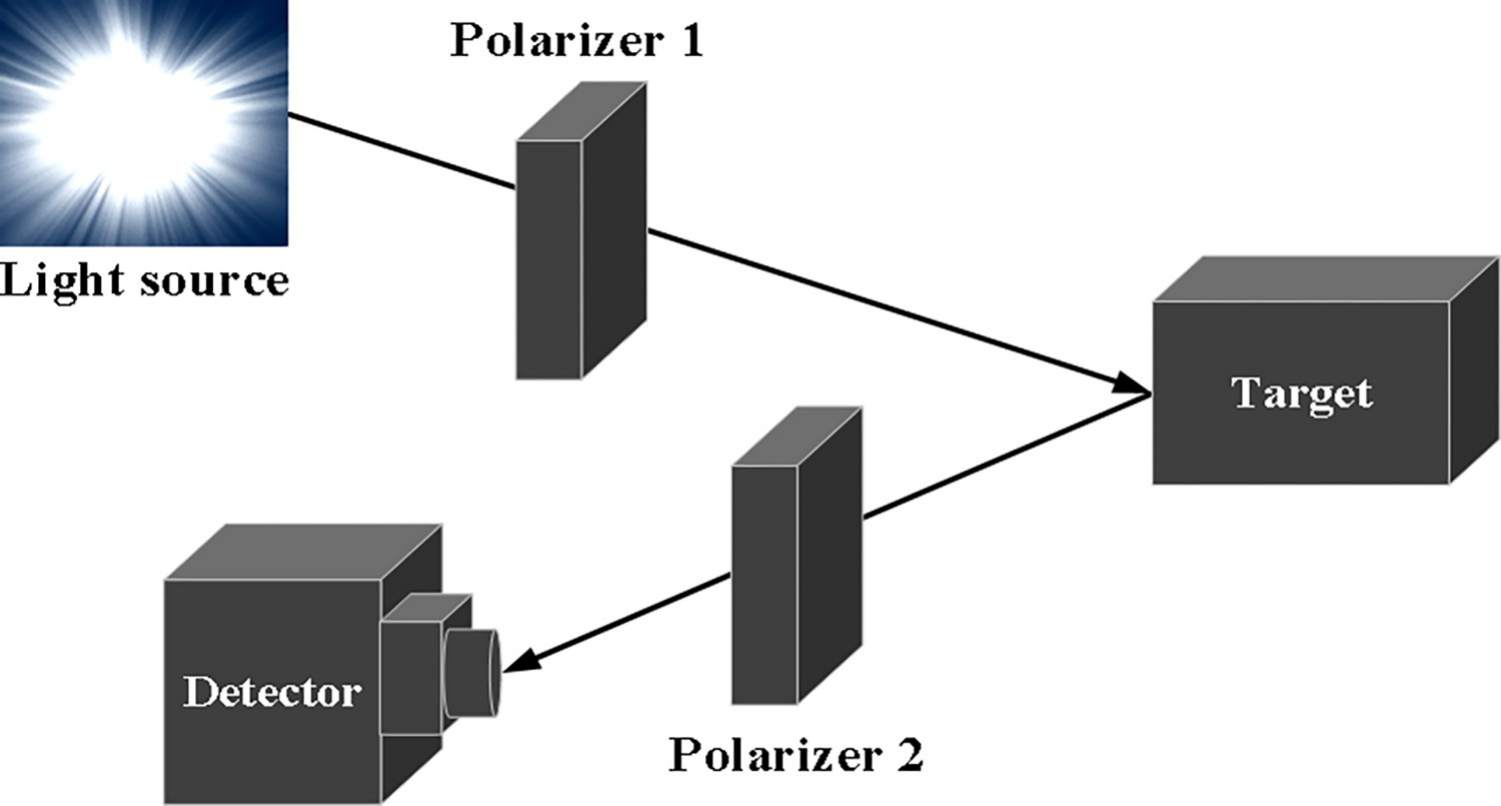
Schematic diagram of the polarization target detection principle.

The principle of polarized target detection is shown in Fig 2. Light emitted from the source passes through polarizer 1, becoming linearly polarized light. After being reflected by the target, the light travels back and reaches the photosensitive surface of the photodetector through polarizer 2. The photodetector then converts the intensity of the reflected light into corresponding electrical current or voltage signals. By rotating the polarizer 2 to 0°, 45°, 90° and 135°respectively, the electrical current or voltage signals can be measured at each angle.

The current detected by the photodetector when the polarization filter is set to 0°, 45°, 90° and 135° respectively is *i*_0°_、*i*_45°_、*i*_90°_、*i*_135°_. Since the current signal outputted by the photodetector is proportional to the intensity of the received signal light, the following relationship holds:

$$\{\begin{array}{c} i_{0^{{}^{\circ}}}=k\cdot I_{0^{{}^{\circ}}}\\ i_{45^{{}^{\circ}}}=k\cdot I_{45^{{}^{\circ}}}\\ i_{90^{{}^{\circ}}}=k\cdot I_{90^{{}^{\circ}}}\\ i_{135^{{}^{\circ}}}=k\cdot I_{135^{{}^{\circ}}} \end{array}$$ (9)

In Eq (9), the symbol ‘k’ represents the current conversion factor. Substituting Eq (9) into Eq (8) gives:

$$DOP=\frac{\sqrt{{\left(i_{0^{{}^{\circ}}}-i_{90^{{}^{\circ}}}\right)}^{2}+{\left(i_{45^{{}^{\circ}}}-i_{135^{{}^{\circ}}}\right)}^{2}}}{(i_{0^{{}^{\circ}}}+i_{90^{{}^{\circ}}})k}=\frac{\sqrt{{\left(i_{0^{{}^{\circ}}}-i_{90^{{}^{\circ}}}\right)}^{2}+{\left(i_{45^{{}^{\circ}}}-i_{135^{{}^{\circ}}}\right)}^{2}}}{\left(i_{0^{{}^{\circ}}}+i_{90^{{}^{\circ}}}\right)}$$ (10)

According to Eq (10), the degree of polarization of the reflected light signal can be obtained utilizing the current signal value detected by the photodetector. When *DOP = 0*, it indicates that the light beam is completely unpolarized (natural light). When *0<DOP<1*, it indicates partially polarized light. When *DOP = 1*, it indicates fully polarized light.

As the light source is a polarized laser source and is intended for use in strong scattering underwater environments, the system demands high precision, accuracy, and sensitivity.

The laser reception section consists of two parts: photodetection and processing of the incident laser information. The photodetection unit receives laser pulse signals, which are then converted into electrical signals by the photodetector (photodiode) and are subsequently outputted as electrical pulse signals after signal amplification and shaping. Finally, signal filtering is applied to achieve signal distinction from various false information contained in the signal. As it involves detecting reflected echoes, the strength of the reflected laser energy is crucial for the device to function properly.

### Overall scheme of the underwater laser target polarization detection system

#### Design of the system scheme

**Fig 3:**
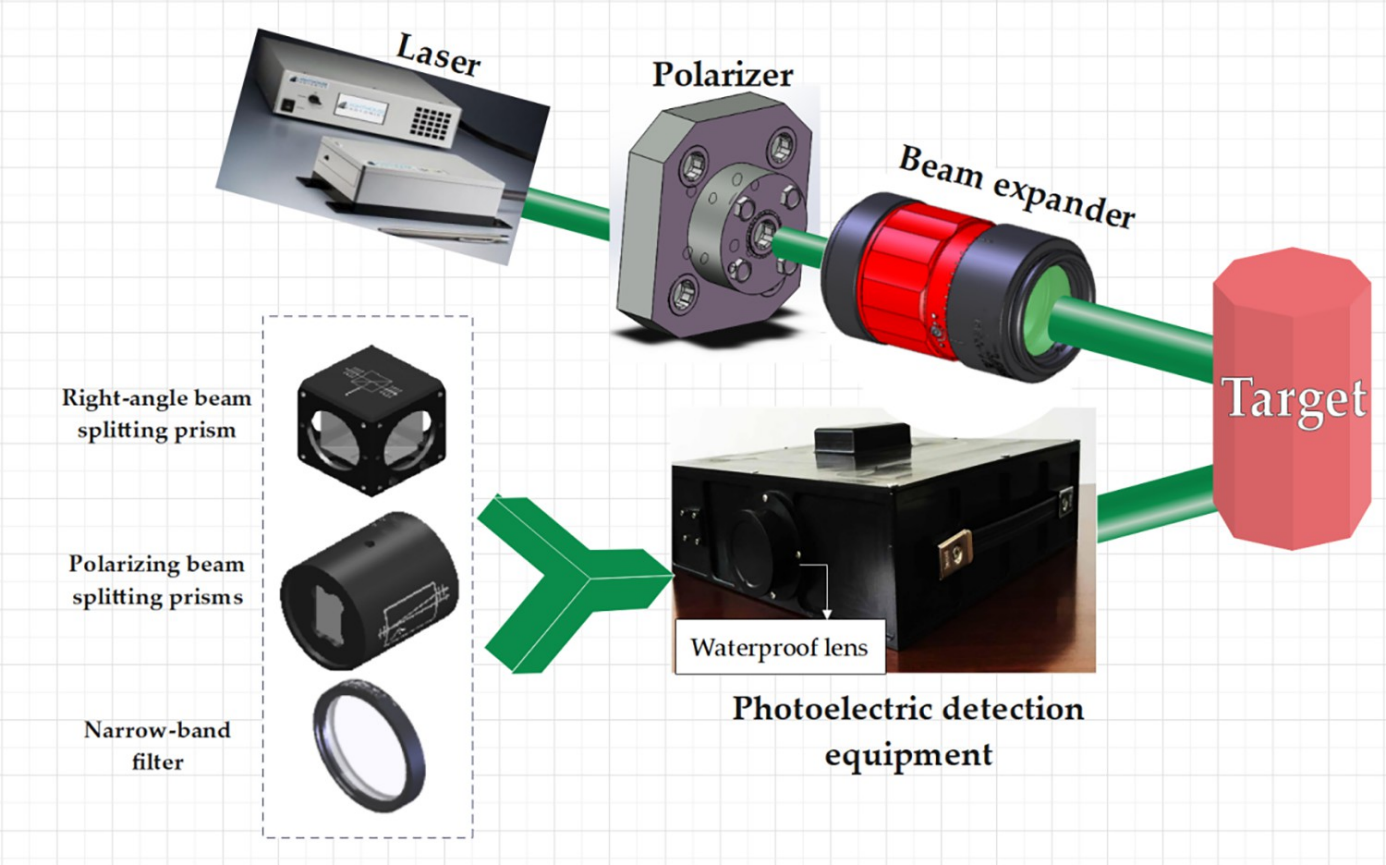
Schematic diagram of laser polarized underwater detection equipment.

The underwater laser target detection system based on polarization utilizes polarization information to differentiate and identify underwater targets. A laser is emitted into the water and directed towards the target being measured. The reflected light is received by photoelectric detection equipment, which is equipped with an optical system. The light is then received by four detectors and the polarization information of the measured object is obtained. The schematic diagram of the system measurement process is shown in Fig 3. The core component of the polarization-based underwater laser target detection system is the photoelectric detection equipment within the dashed box. The main function of photoelectric detection equipment is to receive reflected light from underwater targets, correct optical aberrations, split light, change polarization angles, and collect polarization light information.

Due to the weak natural light in water, a pulsed laser is used for active illumination. The laser emits light that is directed towards the target and is then reflected back to the photoelectric detection equipment. Through the optical system, the light finally reaches the detectors, completing the entire process.

### Laser selection

In the range of 470nm to 580nm, which corresponds to the optical transmission window in seawater, 532nm lasers are a common type of laser. A 532nm single-band laser source can provide a simpler laser device and detection systems, reducing system complexity and cost. It can also provide higher signal intensity and resolution during the imaging process [20], leading to clearer and more accurate imaging results. Additionally, a single-band laser source system is easier to maintain and operate, reducing the possibility of system failures. On the other hand, a multi-band polarization detector system is more complex, requiring more system adjustments and calibrations, which increases operational and maintenance difficulties. The cost of a multi-band polarization detector system is usually higher and more expensive compared to a single-band laser source system.

Ultimately, we chose the DPS-532-B laser for our underwater target polarization detection system. The specific parameters for this laser are shown in Table 1.

**Table 1:** Parameters of the DPS-532-B laser.

Parameter	Values
**Wavelength (nm)**	532±1
**Single pulse energy**	The maximum value is 16.24mJ
**Energy stability (rms)**	<3%, <5%
**Pulse time**	10.12ns
**Working mode**	Pulse
**Frequency**	0~10Hz
**Laser head size and enegry**	315×85×65mm,3.5KG
**Power supply size and weight**	308×306×106mm,5.5KG

### Structural design of detection equipment

#### Working principle of detection equipment

**Fig 4:**
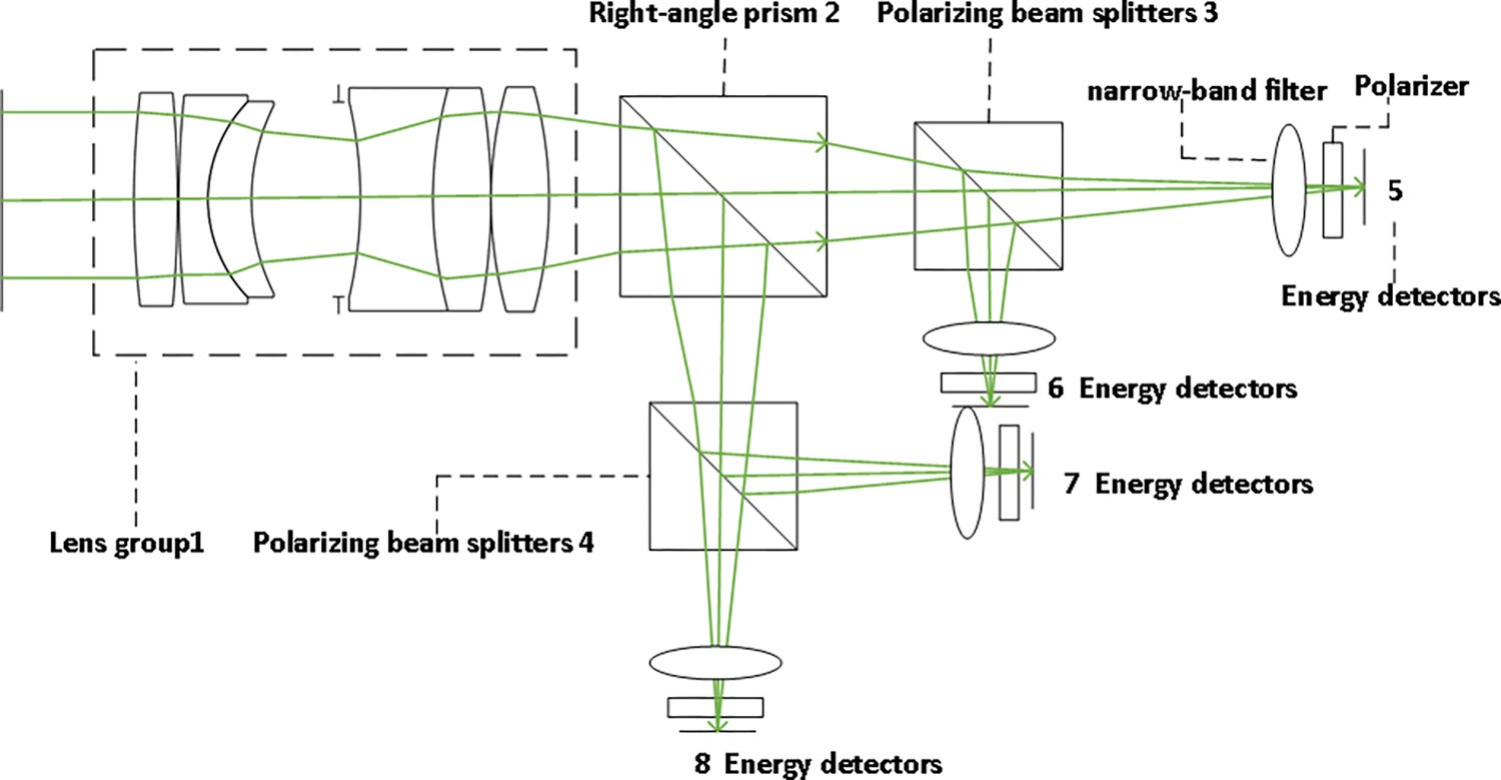
Design of the photoelectric detection unit.

The photodetection device consists of an optical system and an energy detector, as shown in Fig 4, which illustrates the design of the photodetection unit. In the photodetection unit, we have selected a detector with a photosensitive area of 10mm×10mm. To achieve more accurate detection and reduce the laser echo spot, an optical system is employed. Firstly, lens group 1 is installed to optically converge the echo and adjust the light path, ensuring that the laser echo accurately enters the photosensitive surface of the detector. This design utilizes the method of amplitude division to measure Stokes parameters. The incident light is split into two orthogonal beams through a right-angle prism 2. Then, polarized light at 0°, 45°, 90°, and 135° is obtained through polarizing beam splitters 3 and 4, respectively, and enters their respective energy detectors 5 6 7 8. The detectors collect weak electrical current signals that need to be converted into voltage signals. After amplification by an amplification circuit, the amplified voltage signals are converted into digital signals by an analog-to-digital converter (ADC) and then processed further by an FPGA processor. Finally, using Eq (10), the degree of polarization can be calculated, allowing the extraction of polarization elements from the target for detection. Additionally, a polarizer and a 532nm narrowband filter are added in front of the detector to verify whether the light is polarized and to filter out some background light, allowing the reception of laser signals within specific optical windows and reducing noise interference, thus improving the detector’s capability to extract valid signals.

#### Optical design of the photoelectric detection unit

The optical system mainly consists of optical receiving windows, filters, polarizers, and other components. Adding filters to the optical windows can effectively filter out some background light, allowing the reception of laser signals within specific optical windows and, thus, a improve the detector’s ability to capture valid signals.

**Fig 5:**
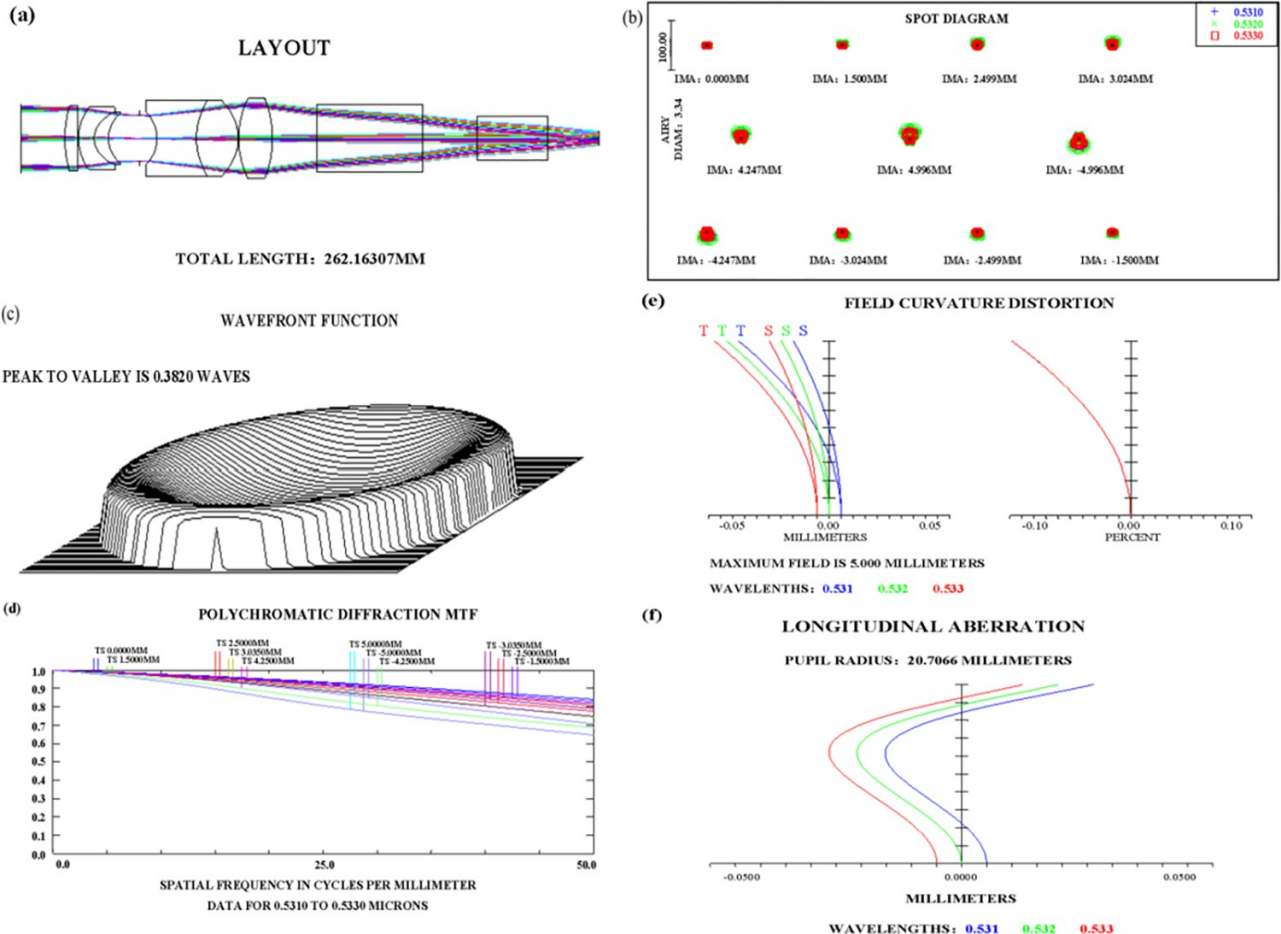
Optical design image quality curve. (a)2D structural diagram, (b)Spot diagram, (c)Wavefront diagram, (d)MTF diagram, (e)Field distortion, (f)Longitudinal aberration.

For imaging requirements, we have adopted a telephoto imaging optical system structure, as depicted in its two-dimensional structure in Fig 5(A). As shown in Fig 5(D), the Modular Transfer Function (MTF) curve of the system indicates that even at 50 line pairs (corresponding to a pixel size of 10μm), the MTF values at various field angles are all greater than 0.5, approaching the diffraction limit. This shows that the system has excellent image quality and high-resolution images. Fig 5(B), 5(C), 5(E), and 5(F) represent the optical system’s array diagram, wavefront map, field distortion map, and spherical aberration, respectively.

#### Circuit design of photoelectric detection unit

When designing the circuit for the photodetection unit, we considered the small energy of the detected echo and the requirement for response speed. Consequently, we plan to use a PIN photodiode to receive the reflected energy of the laser. After reviewing several types of photodetectors, we have selected the PIN photodiode S2387-1010R as the photodetector for its specific characteristics, as shown in Table 2 below. The PIN photodiode is an improved type of photodetector that adds an intrinsic layer (formed by an intrinsic semiconductor) to the PN junction of a regular photodiode, thus increasing the depletion layer and reducing the impact of diffusion motion. Hence, the performance of the PIN photodiode is superior to that of a regular PD diode. The improvements manifest themselves mainly in its linearity and sensitivity, which are significantly enhanced while also reducing its own noise. In addition, it operates at low voltage, maintaining overall stability and ease of use [21].

**Table 2:** Characteristic parameters of the PIN detector.

Parameter	Values
**Spectral response range**	0.34~1.1μm
**Rise time**	3μs
**Noise Equivalent Power (NEP)**	3.1×10^-15^W/Hz^1/2^
**Photosensitive area**	10×10mm
**Conversion efficiency**	0.3
**Photosensitivity**	0.58A/W
**Short-circuit current**	95*μA*(*at* 100*lx*)
**Operation temperature**	-20°C~+60°C

From the above parameters, it can be observed that the PIN photodiode has a relatively large photosensitive area, and its spectral response range includes the 532nm laser being measured. The operating principle of the PIN photodiode is based on the addition of an intrinsic layer (I-region) to the PN junction. When light irradiates the I-region, the generated photogenerated charge carriers will rapidly diffuse to both ends of the I-region, and when they reach the PN junction region, they are separated and captured to produce a current, thereby achieving photoelectric conversion. The energy detection limit of this photodiode is in the range of picoseconds, approximately.

**Fig 6:**
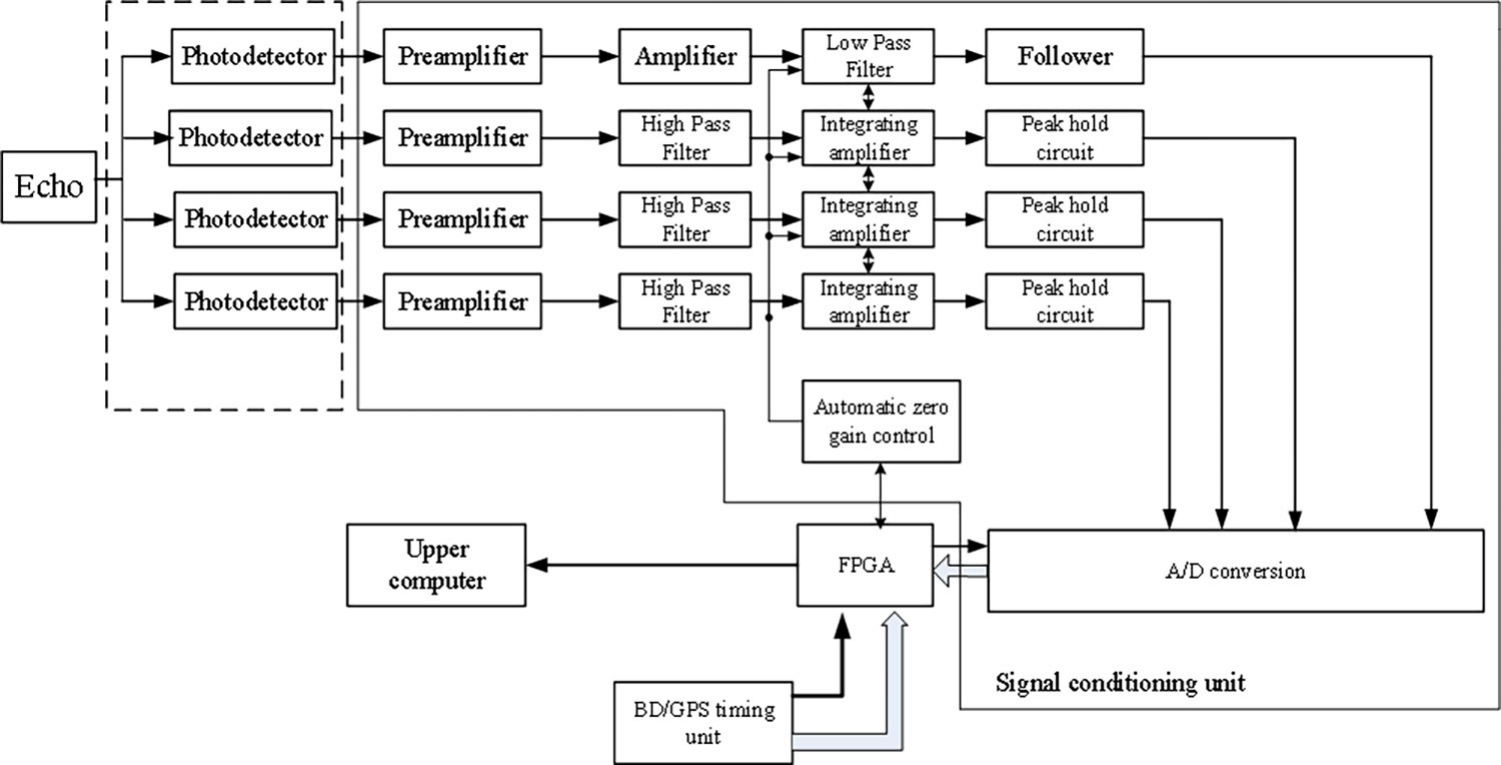
Diagram of the framework for the composition of the plan.

The photodetection device utilizes the photoelectric conversion of the laser echo signals reflected from the target, completing the synchronous sampling and amplification processing of the four-channel signals. The schematic block diagram is illustrated in Fig 6. Typically, the resolution of the ADC is based on the full-scale voltage. To fully utilize the resolution of the ADC, a series of measures such as amplification, attenuation, and filtering are required to bring the analog input signal close to the input range and thus maximize the resolution of the ADC device, enhancing the accuracy of the acquisition circuit. Therefore, the core of the data acquisition signal conditioning circuit lies in the amplification, attenuation, and filtering circuits. After receiving the pulse laser signal, the photodiode obtains the corresponding current value. Due to the short duration of the pulse laser in the nanosecond range, it is necessary to design a signal conditioning circuit that can suppress noise, exhibit high sensitivity, and provide rapid response to the current signal, in order to meet the requirements of the subsequent system.

The PIN photodiode outputs a current signal, and after the I/V conversion, the resulting voltage signal is still very small. As a result, it is necessary to select a low-noise operational amplifier for the design of the conditioning circuit. The sensitivity of the conditioning circuit has a significant impact on the detection of weak signals. For this purpose, a four-channel high-speed precision operational amplifier OP4354 is selected for the conditioning circuit. Its specific performance parameters are shown in Table 3.

**Table 3:** OP4354 performance parameters.

Performance	Parameter
**High pressure swing rate**	150V/us
**Noise**	6.5nV/√Hz
**Bandwidth**	100MHz
**0.01% establishment time**	60ns
**Supply Voltage**	±5V或±15V
**Number of channels**	four

The PIN signal conditioning circuit can be mainly divided into four parts:

The first part is the I/V conversion circuit, which completes the conversion of current to voltage. The second part is the amplification of the requirements of the voltage signal to meet the subsequent measurement system for the amplitude. The third part is the low pass filtering circuit. The fourth part is the voltage follower to achieve impedance matching for the subsequent signal sampling unit. The first two stages utilize operational amplifiers integrated into one chip, designed as two I/V conversion and secondary amplification circuits. The final stage is designed as a voltage follower, eliminating errors caused by individual differences among chips.

### Underwater polarization detection experiment

#### Construction of experimental equipment

**Fig 7:**
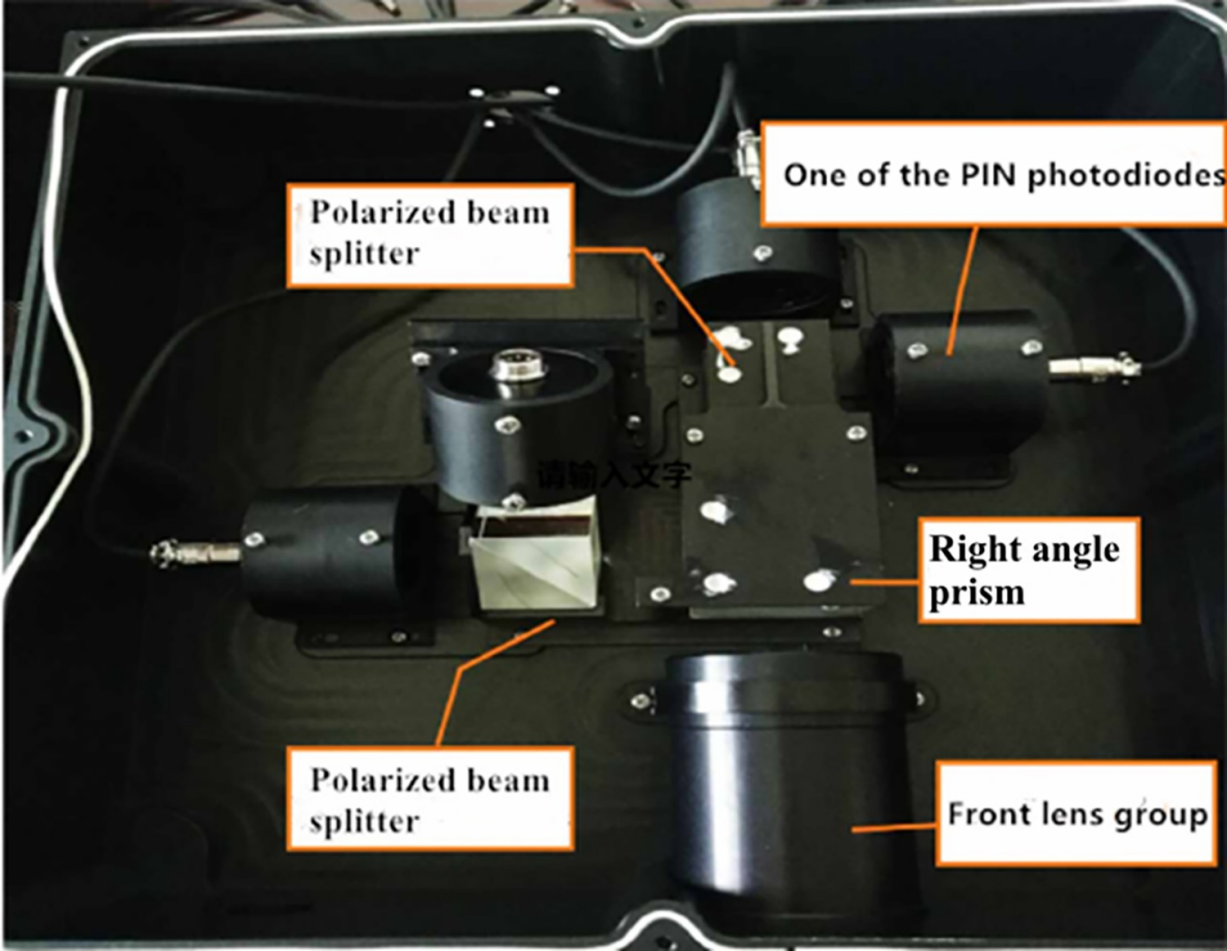
Internal diagram of underwater polarization detector equipment.

After the structural design and processing of the underwater polarized detection system designed in this paper, the physical interior and overall appearance of the underwater polarized detection device are shown in Fig 7.

In the ocean, there exists a ‘water window’ that has good transmittance for blue-green light. Therefore, a 532nm laser is chosen as the light source for the underwater polarized detection device to increase the transmitted light energy. A water tank is used to build the underwater test environment, as shown in Fig 3. Different target objects are placed at different distances in the water tank to simulate the underwater environment. The laser is directed into the simulated seawater environment in the tank, and the energy of the reflected echo from the target surface at 0°, 45°, 90°, and 135° is measured after passing through the water.

**Fig 8:**
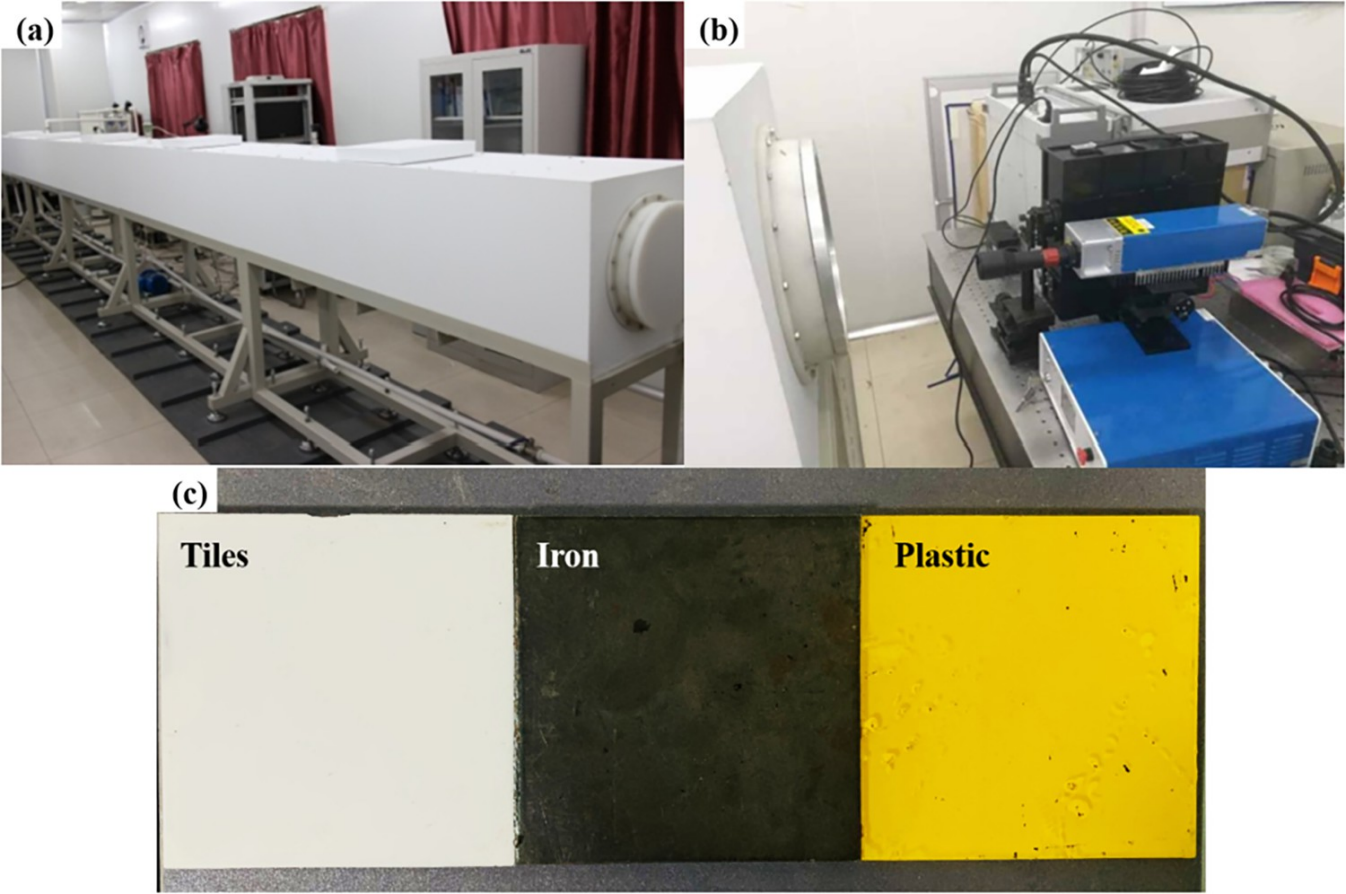
Experimental setup diagram. (a)10m×0.5m×0.5m tank, (b)Experimental system construction, c)Target images of different materials.

In the laboratory, to simulate the water environment at depths of 3 meters, 5 meters, and 10 meters, water is filled into a water tank with dimensions of 10m×0.5m×0.5m, as shown in Fig 8(A). The experimental equipment is set up according to the procedures mentioned, as shown in Fig 8(B), and the experiment begins. Using existing seawater simulation equipment at the experimental university, the underwater environment is simulated. The ends of this water tank are made of glass for light transmission, and there are lids on the sides that can be opened to suspend the objects being measured. In this way, the laser is placed in front of the glass of the water tank to emit laser beams into the tank almost vertically onto the target objects. The underwater polarized detection device is placed on the rear side of the target for measurement.

However, pure water cannot simulate the environment of seawater, so as shown in Table 4, corresponding chemicals are added to the water according to the proportion of various dissolved substances in seawater.

**Table 4:** The proportion of dissolved substances in water.

Material name	Content(%)
**NaCl**	2.3476
**MgCl2**	0.4981
**Na2SO4**	0.3917
**CaCl2**	0.1102
**KCl**	0.0664
**NaHCO3**	0.0192
**KBr**	0.0096
**H3BO3**	0.0026
**SrCl2**	0.0024
**NaF**	0.0003

#### Test results and data analysis

In order to differentiate common underwater targets, a variety of test objects with different materials were selected, including ceramic tiles, iron sheets, and plastic, each with different material properties and varying surface smoothness, as shown in Fig 8(C). Since ceramic tiles, iron sheets, and plastic are all objects with low transmittance and high reflectance, according to references [22, 23], the detectors for these three types of samples were placed in the direction of laser reflection behind the targets for measurement, as shown in Fig 8.

**Fig 9:**
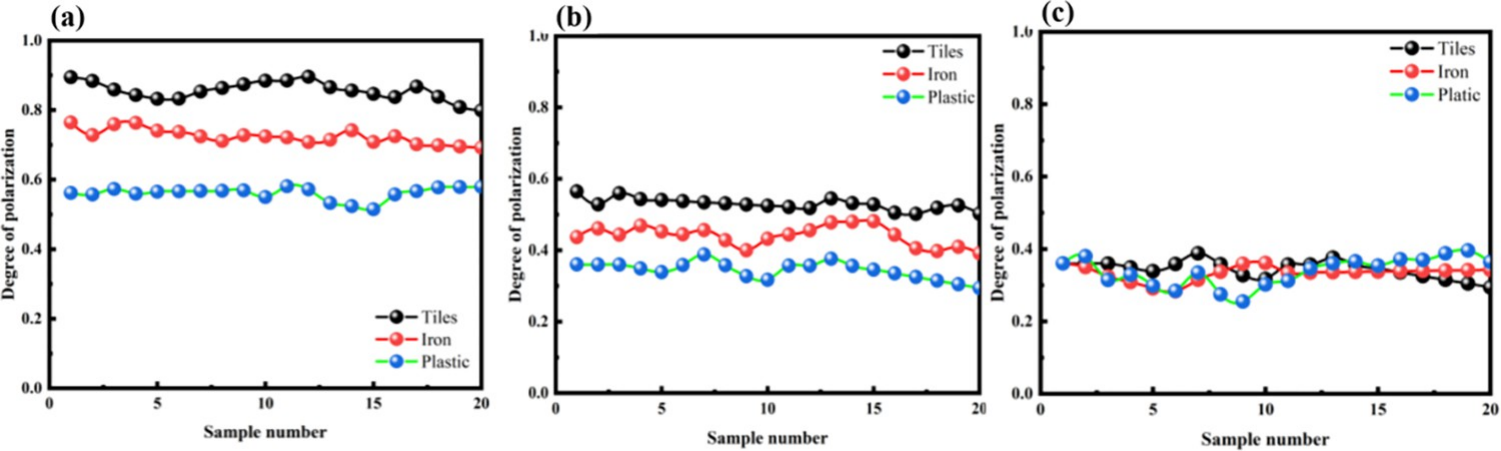
Comparison of the degree of polarization of different objects in water. (a) 3m depth, (b) 5m depth, (c) 10m depth.

Ceramic tiles, iron sheets, and plastic were placed at depths of 3m, 5m, and 10 m for the experiments. To ensure the accuracy of the experimental data, multiple sets of experiments were conducted at each depth to compare the degrees of polarization of different objects in water, as shown in Fig 9. Then, based on the collected polarized light intensity information from the four polarization directions, the degree of polarization and polarization angle were calculated using Eqs (7) and (10). All data were plotted in Table 5.

**Table 5:** Underwater polarization measurement data.

Distance(m)	0°(J)	45°(J)	90°(J)	135°(J)	I	Q	U	DoP	AoP
**Tiles**									
**3.56**	6.45E-11	2.98E-11	8.89E-11	2.1912E-10	2.01E-10	-2.4E-11	-1.9E-10	0.89	41.57
**5.62**	9.43E-10	7.01E-11	1.42E-09	1.3267E-09	1.88E-09	-4.7E-10	-1.3E-09	0.56	34.45
**9.48**	9.63E-10	5.41E-10	1.1E-09	1.2187E-09	1.91E-09	-1.4E-10	-6.8E-10	0.36	39.30
**Iron**									
**3.47**	1.1E-10	5.35E-11	2.45E-10	3.07E-10	3.58E-10	-1.4E-10	-2.5E-10	0.74	30.94
**5.49**	1.84E-10	2.34E-10	3.34E-10	3.68E-10	5.6E-10	-1.5E-10	-1.3E-10	0.45	20.97
**10.06**	1.99E-10	2.02E-10	3E-10	3.65E-10	5.34E-10	-1E-10	-1.6E-10	0.36	29.13
**Plastics**									
**3.35**	7.22E-10	4.6E-10	7.81E-10	1.18E-09	1.57E-09	-5.9E-11	-7.2E-10	0.53	42.67
**5.23**	7.09E-10	6.56E-10	9.39E-10	1.07E-09	1.69E-09	-2.3E-10	-4.1E-10	0.39	30.50
**10.06**	7.73E-10	4.7E-10	7.54E-10	1.05E-09	1.52E-09	1.95E-11	-5.8E-10	0.35	-44.05

From Fig 9, it can be observed that the degree of polarization follows the relationship: *DOP*_*T*iles_>*DOP*_*Iron*_>*DOP*_*Plastic*_. The pattern of degree of polarization is similar at distances of 3m, 5m, and 10m. At a distance of 3 m, there is a significant difference in the degree of polarization among different materials. As the distance increases, the polarization degrees tend to approach around 0.36. This phenomenon may be attributed to the elongated shape of the water tank used in the experiment. With increasing distance, the laser undergoes multiple reflections and scattering between the water and the tank walls, resulting in a more consistent depolarization effect in the end. On the other hand, at closer distances, the primary factor influencing the polarization degree is the polarization characteristics of the target reflections, leading to larger differences in polarization degree between different materials.

**Fig 10:**
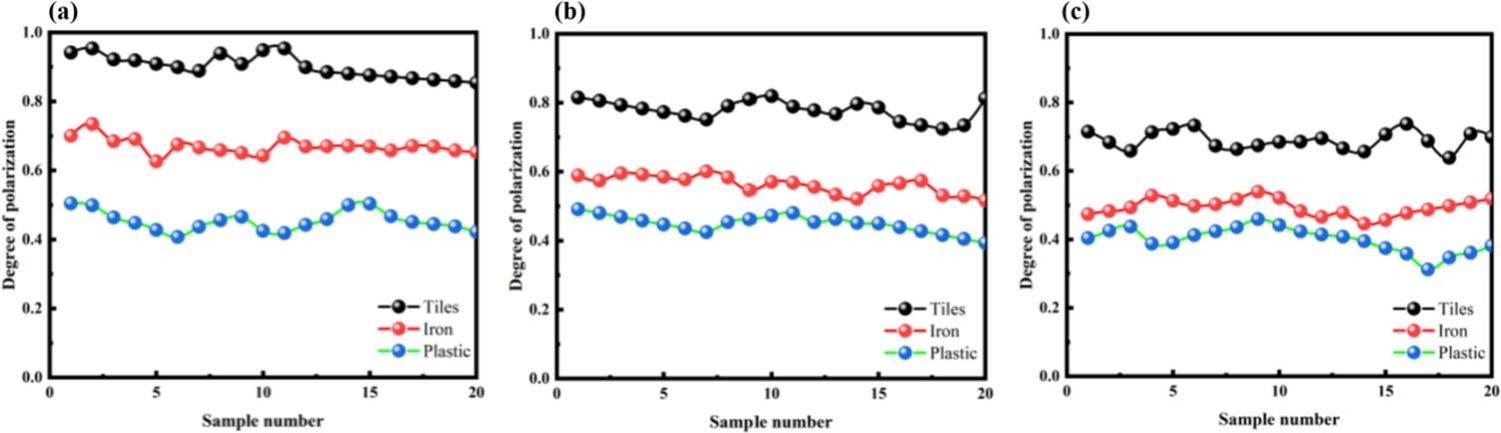
Comparison of the degree of polarization of different objects in the air. (a) 3m depth, (b) 5m depth, (c) 10m depth.

To further validate the reliability of the measurement method for the detection device, the water in the testing environment was drained, and the polarization degrees of the three types of targets were measured repeatedly in an air medium. The objects were placed at distances of 3m, 5m, and 10m for experiments. Multiple sets of experiments were conducted at each depth to compare the polarization degrees of different objects in the air, as shown in Fig 10. The measured and calculated polarization degree results are presented in Table 6.

**Table 6:** Air polarization measurement data.

Distance(m)	0°(J)	45°(J)	90°(J)	135°(J)	I	Q	U	DoP	AoP
**Tiles**									
**3.56**	7.01E-11	3.3E-11	1.98E-10	2.45E-10	2.73E-10	-1.28E-10	-2.12E-10	0.90	29.48
**5.62**	1.83E-09	9.7E-10	2.48E-09	3.34E-09	4.31E-09	-6.43E-10	-2.37E-09	0.77	37.40
**9.48**	2.51E-10	2.46E-10	3.8E-10	5.71E-10	7.24E-10	-1.29E-10	-3.26E-10	0.69	34.22
**Iron**									
**3.47**	8.92E-11	4.49E-11	7.47E-11	2.75E-10	2.42E-10	1.44E-11	-2.30E-10	0.72	-43.20
**5.49**	1.43E-09	7.47E-10	1.98E-09	2.52E-09	3.33E-09	-5.53E-10	-1.77E-09	0.60	36.33
**10.06**	2.19E-10	2.3E-10	3.45E-10	5.11E-10	6.52E-10	-1.26E-10	-2.81E-10	0.47	32.90
**Plastics**									
**3.35**	6.95E-11	3.53E-11	5.73E-11	2.41E-10	2.02E-10	1.22E-11	-2.06E-10	0.58	-43.31
**5.23**	1.44E-09	7.53E-10	2E-09	2.56E-09	3.37E-09	-5.58E-10	-1.80E-09	0.49	36.41
**10.06**	2.51E-10	2.45E-10	3.8E-10	5.71E-10	7.24E-10	-1.29E-10	-3.25E-10	0.40	34.17

Based on Fig 10, it can be observed that the order of polarization degree for the three tested targets is consistent with the trend observed underwater. Because of the different refractive indices of water for different polarization directions of light, it is possible that the measured polarization degrees in water are relatively lower. On the other hand, in air, as there is no significant birefringence effect, the measured polarization degrees tend to be relatively higher.

**Fig 11:**
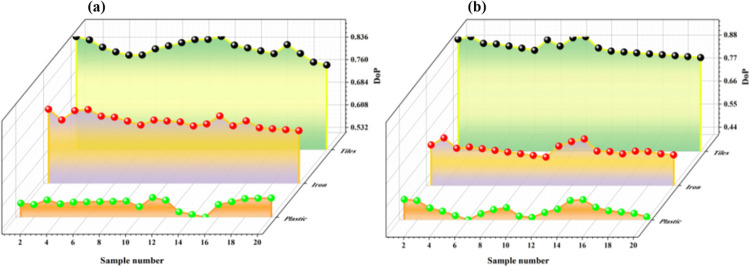
Compare the polarization degree of 3m in different environments. (a) 3 meters underwater depth, (b) 3 meters of air depth.

From Fig 11, it can be observed that the polarization degrees of each material in different media are relatively similar. When analyzing the reasons for the differences in degree of polarization, we see that plastic surfaces are rougher with lower reflectivity, resulting in lower degree of polarization. The iron plate is made of a metal material with a smoother surface and a smaller depolarization effect, leading to a higher degree of polarization. Ceramic tiles, on the other hand, are non-crystalline materials with no regular molecular arrangement. When light passes through ceramic tiles, the direction of oscillation of light changes randomly, resulting in polarization in multiple directions. Therefore, ceramic tiles have a certain transparency for polarized light in different directions, resulting in a higher overall degree of polarization.

In conclusion, using the laser polarization detection device developed in a simulated seawater environment, the tests were conducted on objects with different materials at various depths. The degree of polarization was calculated using the formula based on the test results. The polarization degree results verified that different materials on the surface of the tested targets result in different polarization degrees. This indicates that the experimental system and method can differentiate target materials underwater in terms of detection.

### Comparative experimental verification

For the sake of better validate the laser polarized underwater detection equipment developed in the article, we added a set of polarization camera experiments equipped with Sony IMX250 chips. The specific parameters are shown in Table 7.

**Table 7:** Polarization camera performance specification.

Interface	USB 3.0
Resolving power	2448(H) × 2048(V)
Frame rate	79 fps
Sensor	2/3",Global Shutter
	Sony IMX250MZR CMOS
Pixel size	3.45 μm × 3.45 μm
Pixel depth	8bit、10bit
Spectrum	polarized
Exposure time	20μs~1s

**Fig 12:**
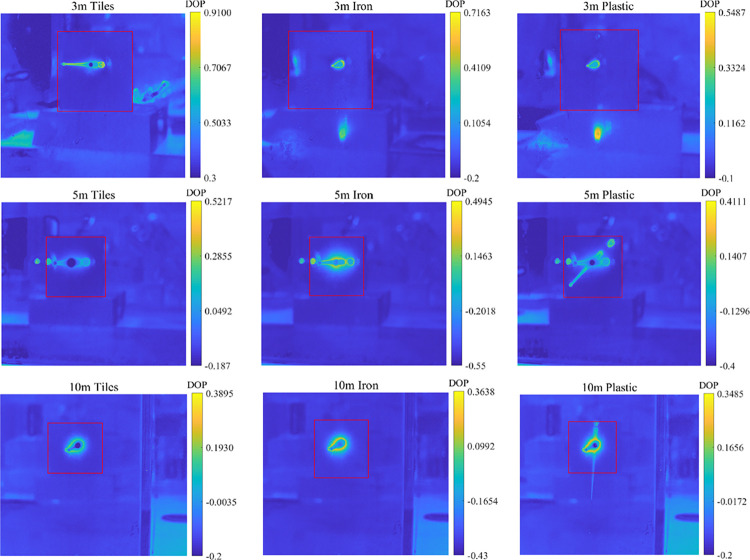
Underwater measurement data.

In order to further verify the reliability of the measurement method of the detection device, we added a set of polarization imaging experiments on tiles, iron, and plastic loaded with Sony IMX250 chips. The degrees of polarization of three tested targets were repeatedly measured in water and placed at 3m, 5m, and 10m, respectively. The degrees of polarization of different underwater objects were compared as shown in the following Fig 12. The measured and calculated polarization degree results are shown in the right part of the figure:

From the above figure, it can be seen that the degree of polarization follows the relationship: *DOP*_*T*iles_>*DOP*_*Iron*_>*DOP*_*Plastic*_. The pattern of degree of polarization is similar at distances of 3m, 5m, and 10m.

**Fig 13:**
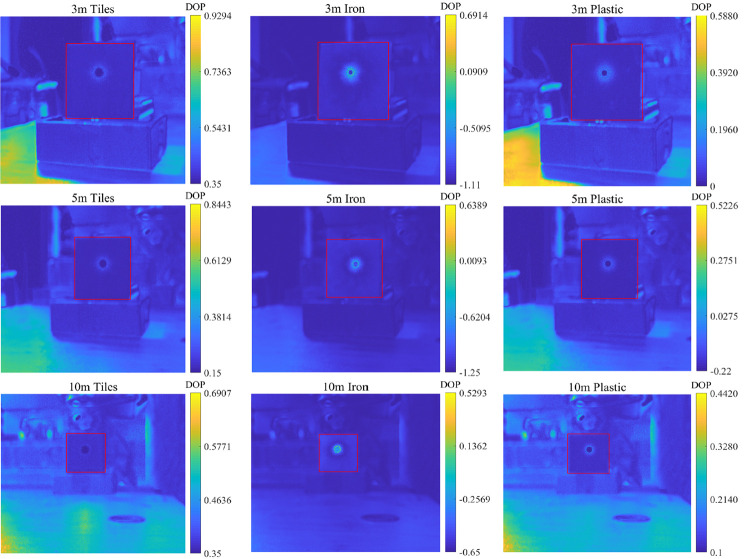
Air measurement data.

In order to verify the integrity of the experiment, we drained the water in the testing environment and repeated measurements of the polarization degree of the three tested targets in an air medium. The experiments were conducted at 3m, 5m, and 10m, respectively. To ensure the accuracy of the experimental data, multiple sets of experiments were conducted at each depth to compare the polarization degree of different objects in the air, as shown in Fig 13. The measured and calculated polarization degree results are shown in the right part of the figure:

From Fig 13, it can be seen that the polarization degree of the three tested targets is arranged in the same order as the trend of underwater testing, with *DOP*_*T*iles_>*DOP*_*Iron*_>*DOP*_*Plastic*_, which is consistent with the conclusion given in this article. Finally, we provide the difference analysis between the measurement data of a polarization camera equipped with Sony IMX250 chip and the measurement data of the laser polarization underwater detection device designed in this paper, as shown in the table below.

According to Table 8, we can see that in the underwater testing experiment, the difference between the measurement data of the polarization camera equipped with Sony IMX250 chip and the measurement data of the laser polarization underwater detection device designed in this paper is within 0.1, and the values of the two are very close with small errors, which verifies the reliability and accuracy of the laser polarization underwater detection device designed in this paper.

**Table 8:** Analysis of differences in underwater measurement data.

	Difference		
	3m	5m	10m
Tiles	0.0139	0.0076	0.0454
Iron	0.0079	0.0540	0.0302
Plastic	0.0123	0.0670	0.0106

Table 9 shows the difference analysis between the measurement data in air, both within 0.1. From this, it can be seen that the data measured by the laser polarized underwater detection equipment designed in this article has high credibility and good reliability.

**Table 9:** Analysis of differences in measurement data in the air.

	Difference		
	3m	5m	10m
Tiles	0.0275	0.0657	0.0006
Iron	0.0207	0.0756	0.0352
Plastic	0.0134	0.0740	0.0425

This can verify the conclusion given in this article. The laser polarization detection device developed in this article is used to test objects of different materials at different depths in a simulated seawater environment. Based on the test results, the degree of polarization is calculated using a formula. The degree of polarization results verify that the surface material of the tested target is different, and the polarization degree is different. This can demonstrate that the experimental system and method can distinguish target materials in underwater detection.

## Discussion

**Fig 14:**
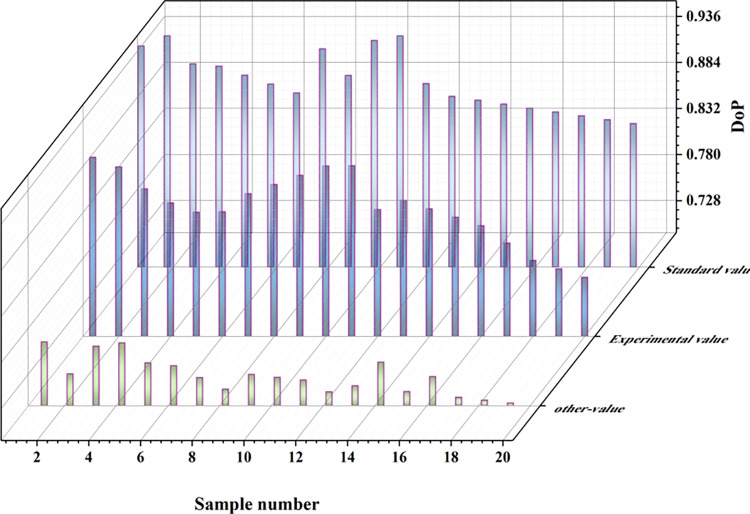
Comparison experiment result chart.

In this study, to rigorously validate the accuracy of our designed underwater laser polarization detection device, we compared it with other underwater polarization detection devices. Reference [24] provided measurement data for the SALSA camera, a commonly used device for underwater target detection. We use these data as a reference standard and compare the precision of our designed underwater laser polarization detection device with that of other researchers’ devices [25]. To better compare the data differences between the two methods and the reference standard, we chose to calculate the root mean square error (RMSE) for both measurements to assess the accuracy of the results. The RMSE is the square root of the ratio of the sum of the squares of the deviations between the observed values and the true values to the number of observations. In practical measurements, the number of observations is always limited, and the true value can only be replaced by the most reliable (best) value. The RMSE is very sensitive to large or small errors in a set of measurements, and thus it can effectively reflect the precision of the measurements. A smaller RMSE indicates higher measurement accuracy. The RMSE for our designed detection device and that of other researchers was calculated using Eq (11). Fig 14 clearly presents the comparison of the results of these three methods, and the specific data are shown in Table 10.

**Table 10:** Comparison results of root mean square errors of three targets.

	Tiles	Iron	Plastic	RMSE
**Standard**	0.801	0.710	0.460	
**This paper**	0.845	0.625	0.420	5.67%
**Others**	0.60	0.417		54.35%

This comparative analysis contributes to a thorough evaluation of the accuracy and precision of our designed underwater laser polarization detection device.


$$RMSE=\sqrt{\frac{\sum_{i=1}^{n}{\left(X_{obs,i}-X_{\mathrm{mod}el,i}\right)}^{2}}{n}}$$ (11)

## Conclusions

Building upon the research background of underwater laser polarization detection devices, we investigated the characterization of polarized light, methods for describing polarization degree, and the approach of measuring polarization degree using the division-of-amplitude method. We determined the detection principle of the system. Additionally, the following conclusions can be drawn. Using the underwater laser polarization detection device developed in this paper, we conducted tests on objects with different materials at various depths in underwater environments. On the basis of the test results, we calculated the polarization degree using the provided formula. By comparing the RMSE of the designed laser polarization detection device with that of the traditional SALSA polarization camera and the stripe tube detection device designed by another researcher, we found that the RMSE of the three targets that used the designed underwater detection device in our study was 5.67%. This suggests that the designed underwater laser polarization detection device offers better accuracy and can distinguish objects with different materials, making it suitable for polarized detection of underwater targets. The research has proven the feasibility of the developed underwater laser polarization detection device. Through experimental studies on laser-based underwater target polarization imaging detection, we achieved polarization imaging of targets with different materials. The current designed underwater detection device enables the analysis of the polarization characteristics of underwater targets, providing a solution to the technical challenges of precise detection and identification of underwater targets in complex environments.
